# Psychoeducation for Patients with Fibromyalgia: A Systematic Review

**DOI:** 10.3390/healthcare9060737

**Published:** 2021-06-16

**Authors:** Lizzette Gómez-de-Regil

**Affiliations:** Hospital Regional de Alta Especialidad de la Península de Yucatán, Mérida 97130, Mexico; gomezderegil@gmail.com

**Keywords:** fibromyalgia, psychoeducation, review, rehabilitation, musculoskeletal

## Abstract

This systematic review presents a brief and comprehensive summary of psychoeducational programs that have been designed for and tested in patients with fibromyalgia, with a focus on the features of the interventions and their main results regarding the patients’ clinical outcome. A bibliographical search performed in PubMed, Web of Science, Scopus, Cochrane Library and PsycInfo targeted publications, related to the terms “fibromyalgia” and “psychoeducation.” Information regarding the studies’ designs, age criterion, sample size, sex distribution and mean age of participants, and assessment time points, was recorded. If applicable, group distributions along with the main results regarding the patients’ clinical outcome, and features of the psychoeducational interventions were registered. Initial search eliciting 77 citations reduced to 10 relevant papers. Most of the reports come from two research projects from Spain (*n* = 6), worked with adult samples (*n* = 9), and most participants were females (88%–98%). Interventions reported significant improvements in the patients’ clinical outcomes in measures of pain intensity, fatigue, sleep quality, depression, anxiety, functional ability cognitive impairment, and quality of life. Results show not only that psychoeducational programs for the treatment of fibromyalgia are feasible, but also that they can be effective for managing physical and emotional symptoms, in particular, pain and depression.

## 1. Introduction

Fibromyalgia (FM) is a chronic pain condition that primarily, though not exclusively, involves the musculoskeletal system. FM is characterized by chronic widespread pain, fatigue, stiffness, poor sleep quality, depressive symptoms, and cognitive problems (e.g., attention, concentration, learning) [1,2]. Average prevalence in the general population of various countries was estimated in 2.7%, ranging from 0.4% to 9.3% [3]. A recent review of literature [4] found a world prevalence in the general population between 0.2 and 6.6%, in women between 2.4 and 6.8%, in urban areas between 0.7 and 11.4%, and in rural areas between 0.1 and 5.2%. In Mexico, FM is the fifth most common musculoskeletal disorder in the general population, following osteoarthritis, back-pain, rheumatic regional pain syndromes, and rheumatoid arthritis, with an estimated prevalence of 0.68% [5]. FM is more prevalent in women (3:1 ratio), people older than 50, with low education level, with low socioeconomic status and living in rural areas [3].

Although most patients with FM are able to function with little disability, the condition implies significant costs to both the individual and the society, as the patient faces simultaneously limitations for work income and the costs of treatment. Wassem and Hendrix [6] reported that a patient with FM faces an annual average costs an $4533 USD, including $3813 USD of direct costs (related to payments for physician and nurse practitioner services, prescription medications, over-the-counter drugs, and nutritional supplements) and $720 USD of indirect costs (time spent by patients and other caregivers as they attempt to treat their illness). Moreover, direct and total costs were found significantly related to well-being, pain, and severity; whereas indirect costs were significantly associated with pain and severity.

The classification and diagnostic criteria for FM have evolved through the past decades [1,7]. Currently, the criteria proposed by the American College of Rheumatology [8] is the most widely used, and it considers the widespread pain index (WPI) in combination with a symptom severity score (SSS) (WPI ≥ 7 and SSS ≥ 5 or WPI 4–6 and SSS ≥ 9), requiring for a diagnosis of FM the presence of generalized pain in at least 4 of 5 regions and the presence of symptoms for at least three months.

Despite the continuous research, the pathophysiology of FM remains incompletely understood [9,10]. As the etiopathogenesis, diagnostic criteria and classification criteria of FM are still debated, so are the treatment strategies. Pharmacological treatment of FM relies mostly on the three drugs that have been approved for this purpose by the FDA (Food and Drug Administration of the United States): pregabalin (a gabapentinoid blocking calcium channels), duloxetine, and milnacipran (both are serotonin-noradrenaline reuptake inhibitors). However, the use of other types of medication, mainly antidepressants (e.g., tricyclic antidepressants, selective serotonin reuptake inhibitors, cyclobenzaprine), is constantly being tested [2,11]. Regardless all efforts, the efficacy of pharmacological treatment is rather modest, often leading to voluntary discontinuation of medication intake as patients with FM perceive little amelioration [11,12].

Current clinical guidelines [13,14,15] promote a multidisciplinary approach to improve the effectiveness of interventions for the treatment of FM. In addition to medication, non-pharmacological complimentary options have been proposed, including psychotherapy, exercise, biofeedback, hydrotherapy, meditation, and acupuncture, among others [2,13]. Reviewing the available evidence [14], the three best-studied non-pharmacological therapies are education, cognitive behavioral therapy (CBT), and exercise, and results suggest that the magnitude of their treatment often exceeds that for medications, particularly in functionality improvement.

Psychological distress is not uncommon when facing a chronic illness and this might be even more overwhelming when information about the causes, prognosis, and treatment of the condition is limited, as it is the case of FM. Although the direction of the effect cannot be defined, there is ample evidence of unfavorable mental health states in patient with FM. Research has found a high prevalence of psychiatric comorbidities (mostly depression, anxiety, borderline personality, obsessive-compulsive personality, and post-traumatic stress disorder), high levels of negative effect (neuroticism, perfectionism, stress, anger, and alexithymia), a significant tendency to a negative self-image and body image perception, low self-esteem, and perceived self-efficacy [16]. FM exposes the individual to continuous pain, both physical and emotional, antidepressant drugs and psychological interventions have helped to ameliorate these concurrent symptoms, suggesting a link between the physical and the psychological dimensions of the syndrome [17]. The common presence of negative mental states in FM might be interpreted as a consequence of living with chronic pain and other disruptive symptoms; on the contrary, mental disturbances, particularly depression, can be seen as risk factors triggering FM onset or as the underlying cause of FM as a psychosomatic disorder. Furthermore, it has also been argued that depression and FM might be both part of the same spectrum of disorders, sharing underlying pathophysiological pathways, such as hypothalamic–pituitary–adrenal axis dysfunction and/or inflammatory dysregulation [18].

From a psychological perspective, health researchers and professionals have proposed two main types of interventions, psychotherapy and psychoeducation, aiming at providing the patients, particularly those with a chronic illness, with skills for a better self-management, enhancing treatment adherence, preventing relapses/aggravations, and improving the quality of life. Psychotherapy relies on the assumption that the person seeking help acknowledges that is going through a life situation that exceeds his/her abilities to control emotions and find a satisfactory solution. With the guidance of the psychotherapist, the patient must find healthy strategies to eliminate or control troubling symptoms, so that he/she can function better and increase healing and well-being. Regarding the provision of psychotherapy for chronic illnesses, initial sessions might be in group, and in this case the aim would be that patients can identify symptoms of emotional discomfort, share their emotions with other patients in similar conditions receiving immediate feedback, learn strategies to prevent stress reactions that may worsen their physical and emotional states, and seek help if in need. Individual psychotherapeutic sessions should prioritize those patients showing high psychological distress with a possible mental disorder (e.g., depression, anxiety) needed to be clinically diagnosed and treated.

Psychotherapeutic interventions in patients with FM include CBT, guided imagery, hypnosis, acceptance and commitment therapy, and mindfulness, among other approaches [19,20,21]. A recent review [19] on psychotherapy for the management of physical pain in patients with FM found that CBT and guided imagery were the two most commonly used strategies. Moreover, although most studies reported a reduction in pain, this was not always significant and/or lasting. It must be pointed out that research literature shows diversity not only of psychotherapeutic approaches, but also of outcome measures, assessment tools, and sample characteristics, hindering the comparison of results across studies and the reach of deeper conclusions.

The field of psychology has also proposed psychoeducational interventions; they can be seen as the integration of health psychology with behavioral counselling and even psychotherapy [22]. Psychoeducation is defined as “an intervention with systematic, structured, and didactic knowledge transfer for an illness and its treatment, integrating emotional and motivational aspects to enable patients to cope with the illness and to improve its treatment adherence and efficacy” [23]. Psychoeducation can be particularly useful in the management of chronic illnesses, physical or mental, that require long-term treatment. The educational component teaches facts about the physical or psychological condition that is shared by the participants in the group, whereas the behavioral counselling component focuses on emotions, perceptions, coping, relaxation, and self-care [22]. When receiving a diagnosis, patients usually received generic information about the illness; yet, this information might not be enough to help them as they may later find themselves with doubts and fears. Psychoeducation goes beyond the sole practice of informing the patient about the disorder or giving them some basic guidelines on how to manage crises; it consists of three key elements: (1) information about the illness, (2) education in communication skills and problem-solving techniques, and (3) therapeutic support [24]. These three elements must cover at least the following four issues: (1) treatment of the condition, (2) management of the condition, (3) compliance with the medical and psychological regimen, and (4) prevention of progression, exacerbation, or relapse [22].

Psychoeducational sessions may be led by trained health staff (not necessary a psychotherapist) and are provided in small groups. Through psychoeducation the patients, and in some cases also their relatives, can learn about their medical condition, having the opportunity to express their questions and emotions, and letting go of misbeliefs and fears.

Psychoeducation improves health outcomes and reduces healthcare costs with patients with chronic physical diseases, psychological conditions, high utilizers (somatizers) of medical services, and other several categories of patients [22].

Psychoeducation has shown some promising results in patients with FM, improving functional status, management of emotions, perception of pain, and anxiety and depressive symptoms [25]. In a multidisciplinary approach for the treatment of FM, psychoeducation is recommended as an initial step, leaving the possibility of later referral to psychotherapy only if considered necessary [13,15].

FM is a chronic condition demanding patients to adapt to a variety of physical (e.g., pain, stiffness) and emotional (e.g., anxiety, depression) symptoms. Psychology, in the form of psychotherapy or psychoeducation, provides complementary treatment to help patients improve their skills to control negative emotions that may trigger or worsen FM physical symptoms. Systematic reviews have synthesized the results from psychotherapeutic interventions implemented in patients with FM [26], for instance, for the management of physical pain [19], the use of CBT [27], mindfulness [28], or guided imagery and hypnosis [29], and the group [20] or Internet [30] modalities. Psychoeducation programs provide the patient the opportunity to learn about this condition and to develop emotional skills for self-management. To the best knowledge of the author, only one systematic review [25] has been published regarding psychoeducation in patients with FM, summarizing the evidence of statistically significant positive results when comparing its effectiveness with treatment as usual and with other non-pharmacological interventions, also when provided in an online format. Here, this systematic review presents a brief and comprehensive summary of psychoeducational programs that have been designed for and tested in patients with FM with a focus on the features of the interventions and their main results regarding the patients’ clinical outcome. This review might be of interest to professional and researchers working with this population and for patients themselves.

## 2. Materials and Methods

This systematic review was conducted within the Reporting Items for Systematic reviews and Meta-Analyses (PRISMA) statement [31]. Ethical approval was not required for this review. The review methods were established prior to conducting the review.

According to the PICOS (patient/population, intervention, comparison and outcomes) model [32], the review focuses on: (1) population—patients with a diagnosis of FM according to the American College of Rheumatology [8], (2) intervention—psychoeducation, (3) comparators—any type, (4) outcomes—patients’ physical or psychological outcome measures, (5) study design—interventional studies with or without control group(s).

Published articles (all years included) describing psychoeducational interventions for patients with a diagnosis of FM were targeted. The inclusion criteria regarding the manuscripts’ features were: (1) research papers, (2) published in peer-reviewed journals, (3) available in English or Spanish. Additionally, inclusion criteria regarding the studies’ designs were: (4) participants with a primary diagnosis of FM and, (5) a psychoeducational program as an intervention. The exclusion criteria were: (1) not original research (e.g., letters, dissertations, reviews and/or meta-analyses), (2) sample including totally or partially patients with a clinical diagnosis other than FM.

Author performed a bibliographical search in the PubMed, Web of Science, Scopus, Cochrane Library and PsycInfo electronic databases, on 13 October 2020. The terms “fibromyalgia” and “psychoeducation” were entered (Table 1). Following the initial search from the electronic databases and the filtering of duplicated results, the titles and abstracts of the retrieved studies were screened to identify suitable research that met the inclusion/exclusion criteria. The potentially eligible articles were then reviewed in full text to verify their pertinence to the objectives of the study and, if relevant, to record the following information: year of publication, setting of research team, study design, age criterion, sample size, sex distribution and mean age of participants, and assessment time points. If applicable, group distributions along with the main results regarding the patients’ clinical outcome, and features of the psychoeducational interventions were registered.

**Figure 1:**
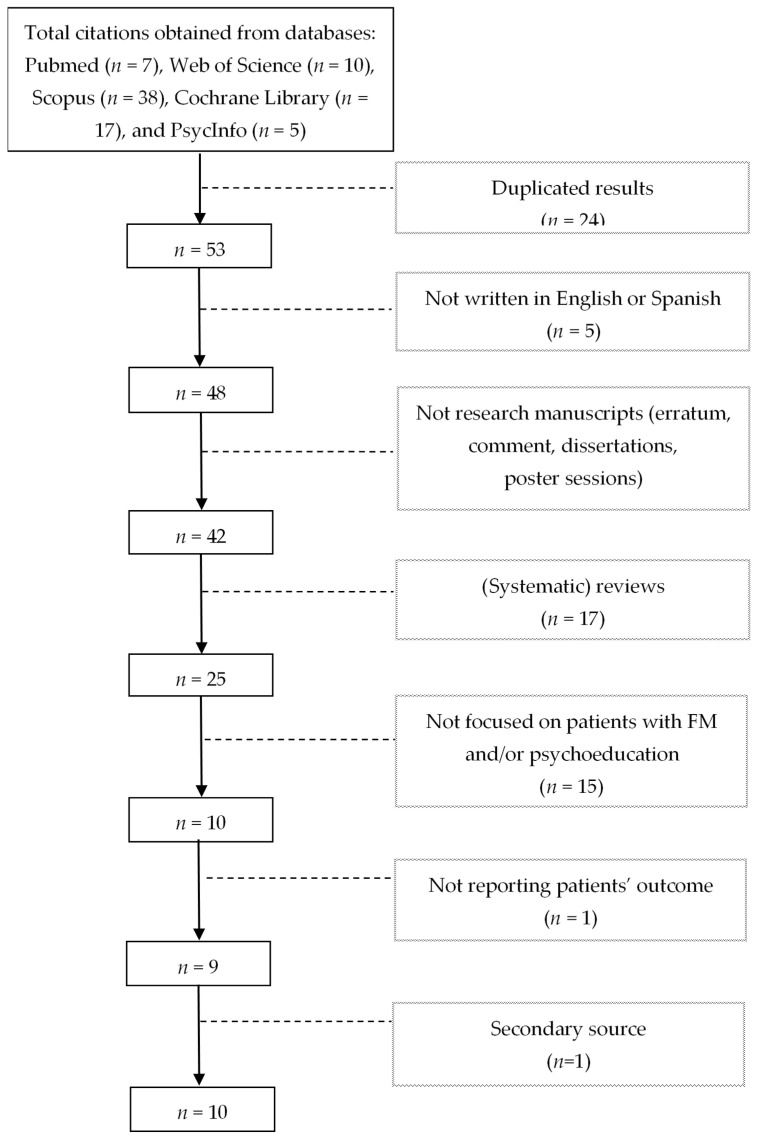
Study flow diagram.

Two researchers collaborated as invited raters to assess the methodological quality of the selected studies, using the quality assessment tools for controlled intervention studies and for pre-post studies with no control group proposed by the United States Department of Health & Human Services [33]. Figure 1 shows the study flow diagram.

## 3. Results

The initial search from the five electronic databases produced 77 results, with 24 duplicates. Applying the selection criteria the list reduced to 25 potential papers. Following, by reading the full content of the manuscripts, 14 were eliminated as they did not focus on patients with FM and/or did not include a psychoeducational intervention. One more article was not considered given that its reported outcomes were not related to patients, but rather on the cost-utility of the intervention. The author obtained one additional article by searching manually through reference lists of the primary studies. Papers reporting study protocols were included, considering that they may provide information regarding the design and the proposed psychoeducational intervention. The final sample (Figure 1) included 10 publications, with 8 original research manuscripts and two study protocols.

Table 2 summarizes the basic features of the selected publications, all in a year ranging from 2006 to 2019. Most of the reports come from two research projects from Spain (n = 6), followed by research from the United States (*n* = 3), and one study from Canada. Most of the studies reported to have received funding for the research project and/or for the authors, providing them with a research contract or with an academic grant. None of the studies reported any competing interests.

Five of the six reported projects were randomized control trials, 3 with a two-arm design and 2 with a three-arm design. Noticeably, a significant percentage of participants were females (mean = 94.5%, range: 88%–98.2%) and adults (mean = 52.3, range: 46.6–55.0), and only one study focused on patients with juvenile FM. All studies compared the patients’ outcome immediately after treatment, and four projects considered follow-up assessments, from one to 12 months after intervention.

Regarding group distribution, two publications reported studies with a single group, one performed with a juvenile sample and another as a pilot study of a planned three-arm randomized control trial. Three projects (reported in 5 publications) relied on a two-arm randomized control trial design, comparing the effect of adding up to usual care an experimental psychoeducational intervention. Two projects (reported in 3 publications) relied on a three-arm randomized control trial design, one combining cognitive behavioral therapy or education with medication and the other comparing treatment as usual to adding up either training in mindfulness-based stress reduction techniques or a psychoeducational program for quality of life improvement, Table 2 and Table 3. The study by Degotardi and colleagues [37] on juvenile FM relied on a single group, integrating patients and their parents to a psychoeducational intervention lasting 8 weeks. Assessments proceeded before and immediately after treatment. It must be mentioned that these authors comment in their publication the performance of previous pilot studies, the first one randomly assigning 15 children to either a CBT intervention group or to a control group receiving therapist attention and relaxation training, and a second pilot study with a single group of 40 children assessed before and after CBT treatment. Authors decided not randomizing as their pilot study data suggested that the inclusion of a control group was ineffective and ethically questionable. Moreover, another important bias was the parents’ willingness and availability to attend the sessions along with their children. As authors reported, even those initially entering the study showed an important attrition rate of 34%, due to difficulties with schedule, accessibility, or insurance coverage, dissatisfaction with the program, need for psychiatric referral or just not returning post-treatment questionnaires. Another study used a single group design [40]; yet, it was performed as a pilot for a RCT [41,42] to be commented later. Three projects relied on a two-arm RCT design. Anderson and colleagues [34] assessed the effectiveness of a multidisciplinary program including CBT/psychoeducational classes, along with exercise, massage, auricular therapy, microcurrent therapy, and nutritional counselling. All participants received standard medical care, while the experimental group received the tested add-on program. The CBT/psychoeducational classes were only a part of phase 1 (out of 3), during the first eight weeks, of the whole program lasting approximately one year. Assessments were made before and after intervention with no follow-up. Bourgault and colleagues [36] followed a two-arm RCT design with a wait-list. The PASSAGE (Programme d’Apprentissage de StratégieS d’Auto-Gestion Efficaces / Training Program of Efficient Self-Management Strategies) program consisted of 9 sessions; the first 8 were held over a period of 11 weeks and the final session 9 was 6 months later to review progress and gain maintenance. Data were collected at baseline, at the end of the intervention, and 3 months later for both groups. Follow-up data at 6 and 12 months after the intervention was also collected from the first group, at the time when the initial wait-list group was receiving the intervention. Luciano and colleagues [38,39,43], the FibroQoL study group, designed a two-arm RCT, comparing usual care to receiving additionally a 9-session psychoeducational program lasting 2 months. Base line, after intervention and at 6 and 12 month follow-ups, data were collected. The FibroQoL research group aimed at testing the effectiveness of ad-hoc designed psychoeducational program implemented in primary care centers to improve the quality of life of patients with FM, and reduce the use of healthcare and social services. Two research projects [35,41,42] presented a three-arm RCT design. Ang and colleagues [35] compared group of patients receiving 8 weekly sessions of phone-delivered CBT (with medication or placebo) or educational information (with medication). Assessments took place at baseline, after treatment and at 21-week follow-up. Authors acknowledge that the absence of a placebo and education control group limits the possible conclusions regarding the interaction of CBT with medication; yet, they considered that, given the established efficacy of the medication (i.e., milnacipran) a fourth arm (double placebo) was neither scientifically necessary nor ethically justified. Pérez-Aranda and colleagues [41,42] compared three groups receiving treatment as usual alone, accompanied with MBSR (Mindfulness-Based Stress Reduction) or with a psychoeducational program. Add-on treatments were provided through 8 sessions, with assessments performed at baseline, after treatment and 12 months later. The objective of the project was to test the efficacy for reducing functional impairments and cost-utility of MBSR; the inclusion of the FibroQoL psychoeducational program as an active treatment comparator was due to its satisfactory previous evidence on these outcomes. As mentioned previously, authors later incorporated both interventions into the MINDSET program [40]. Most projects were RCTs, comparing one or two complementary treatments. It is important to underline that in all cases treatment as usual was provided, including at least rheumatologic medication and appointments, given that psychoeducational programs are meant as complements to usual care, not as substitutes. Moreover, following research ethics, authors guaranteed that the tested programs were offered to all participants, either as members of the experimental group, or once the study was concluded.

Overall, interventions reported significant improvements in the patients’ clinical outcomes in measures of pain intensity, fatigue, sleep quality, depression, anxiety, functional ability cognitive impairment, and quality of life (Table 3).

Psychoeducational interventions lasted 8 or 9 weeks, and although diverse, they share in common the inclusion of educational sessions providing an overview of FM (e.g., diagnosis, symptoms, etiology, and treatments) and skill training sessions for the self-management of emotions related to FM symptoms and the development of healthy habits. Table 4. Anderson and colleagues [34] tested the effectives of adding non-pharmacological treatment to the usual pharmacological treatment. The first phase of the program offered psychoeducational classes, with a focus on CBT coping techniques (e.g., relaxation, problem solving, managing distorted thoughts and feelings). However, the whole program lasting one year included other non-pharmacological approaches, such as exercise, massage therapy, auricular therapy and nutritional counselling. Ang and colleagues [35] organized two groups by the use of CBT with medication (combination therapy) or placebo (CBT monotherapy), and a control group receiving medication along with education (drug monotherapy). The CBT sessions included education only in regard to theories of pain and its relation to automatic thoughts, while providing techniques for symptom management and healthy habits. The drug monotherapy group received educational sessions, neutral with respect to actual problem-solving, regarding cost of chronic pain, acute versus chronic pain, sleep, depression and other mood changes, pain and communication, working with health care providers, and how to make changes. Bourgault and colleagues [36] developed PASSAGE, an interdisciplinary intervention including psychoeducation, CBT techniques and exercise. These three dimensions, although differentiated in the manual content, were presented all together in each session of the program. Degortadi and colleagues [37] presented a program for juvenile primary fibromyalgia syndrome for young patients and their parents. Authors conceptualize the program as a CBT intervention including psychoeducation as one of four modules, along with sleep improvement, pain management, and activities of daily living. Luciano and colleagues [38,39,43], the FibroQoL study group, developed a psychoeducational program for improving the quality of life of patients with FM, including information about the illness, counselling about physical exercise, and training in autogenic relaxation. Pérez-Aranda and colleagues, the EUDAIMON study group, initially considered a Mindfulness-Based Stress Reduction intervention and the FibroQoL psychoeducational program as independent add-ons to treatment as usual [41,42], and later integrated both alternatives into the MINDSET (mindfulness and education) program [40].

Two invited raters independently assessed the quality of the studies (excluding protocols) (results available from author upon request). Interrater reliability by intraclass correlation coefficient (by two-way mixed model and absolute agreement) was 0.90 (95% CI = 0.62–0.91) for the two studies with no control group and 0.94 (95% CI = 0.83–0.92) for the six randomized control trials.

## 4. Discussion

The journey of a patient with FM may be despairing. It is not unusual that the diagnosis of FM is finally given after years of onset and visits to diverse medical specialists. Having a diagnosis may bring relief to the patient; yet, only temporally. Patients with FM may experience frustration and hopelessness, as their symptoms seem unpredictable and fluctuating, resistant to treatment, and in many occasions, discredited by relatives and even by some health professionals.

FM is characterized by continuous physical pain that may range from a mild discomfort to a severe and disabling distress, and it might be accompanied with other common physical (e.g., fatigue, stiffness), cognitive (e.g., fibro fog), and emotional (e.g., anxiety, depression) symptoms. As patients with FM seek attention from physicians, pharmacological treatment for pain is the usual option provided at first [1]. Nevertheless, it is now widely acknowledged that the effectiveness of such treatment alone is moderate [44]; yet, it can be incremented with complimentary options (e.g., education, psychotherapy, and exercise) [14]. Thus, pharmacological treatment, patient education, psychotherapy, and fitness have been proposed as the four pillars in the treatment of FM [1].

Psychoeducation is a treatment option that integrates both, the educational and the psychotherapeutic approaches, providing participants with up to date information regarding the illness (e.g., symptoms, etiology, treatment, and prognosis), training for emotional coping skills and psychotherapeutic support. This review aimed at summarizing information regarding the main features and contents of psychoeducational programs designed for and tested in patients with FM, along with any evidence of a positive and significant effect on the patients’ clinical outcome.

According to the eligibility criteria, the systematic search yielded only 10 relevant publications, from 4 research projects. The number seems limited, considering that current guidelines [13,14,15] strongly recommend psychoeducation as an important element in an optimal multidisciplinary approach for the treatment of FM, and research has shown its promising effects in diverse outcomes such as functional status, sense of fatigue and stiffness, perceived pain and depression, anxiety, and quality of life [25]. It may be the case that psychoeducational content is not commonly presented as an independent program but rather as part of broader interdisciplinary non-pharmacological treatment interventions, as in the selected publications. These publications show that psychoeducation and psychotherapy often overlap and in some cases their names are used interchangeably. Psychoeducation implies giving updated medical information regarding the illness (e.g., risk factors, symptoms, treatment, prognosis), but it goes beyond, targeting other individual psychic elements (e.g., dysfunctional beliefs, emotions and behaviors) influencing the patients’ attitudes toward their illness, that may improve or worsen the adherence to treatment and the severity of symptoms. CBT is the most often used psychotherapeutic approach in the psychoeducation programs. CBT assumes that negative emotions result from dysfunctional ideas framed by the person’s system of beliefs. Thus, CBT sessions help the patients identify those distorted beliefs that may influence the severity of the symptoms, and to concede to behavioral and cognitive changes, substituting dysfunctional schemes [45]. These results show that although psychoeducational programs for patients with FM have basic features in common, their content and arrangement seem quite heterogeneous. This may respond to the different target populations and settings, as health providers and researchers must adapt the interventions to the needs of patients and the circumstances of the locations. Yet, this same condition, along with the diversity of measures, is unfavorable for an integrative interpretation of the results across studies.

Although all the studies recruited patients of both sexes, female participation rates were noticeable higher. Also, all but one of the studies focused on adult samples, and mean age of participants was around 50 years. These results concur with the reported estimations of higher prevalence of FM in females and people older than 50 [3]. Only one study presented a program designed for juvenile patients [37]. This study brings forward some reflections worth mentioning. First, FM may be present since young ages (estimations have reported prevalence between 0.5 in ages 0 to 4 and 6.2 in ages 9 to 15) and following a chronic course it is likely to impact the functional status and the psychosocial development of the youngster [46]. Also, parents play a fundamental role in assuring the patient receives a prompt diagnosis and properly follows treatment. Thus, for this age group, the patients as well as their parents must definitely be considered as recipients of the psychoeducational intervention. Moreover, materials and issues must adapt to the developmental stage of the young patient, as Degotardi and colleagues [37] did in their study. For the age leap 8–11, authors prioritized the self-management while being at school; 12–14, independence from parents and self-monitoring of treatment adherence; and 15–20, assuming personal responsibility for managing FM symptoms, coping with the overwhelming demands of school and other activities, dating, and preparing for the transition to college and independent living.

Sample sizes ranged from 35 (a pilot study) to 216 (two-arm RCT) participants. The studies by Bourgault [36], Luciano [38,39], and Pérez [41] and their colleagues reported sample size estimation and met the minimum required number of participants. The studies by Degotardi [37] and by Pérez [40] and their colleagues had a single group with no reported estimation of sample size. The studies by Anderson [34] and by Ang [35] acknowledged their smaller sample size could have affected the results; yet, only the former verified that an increased sample sized would have not influenced their statistical significance.

Psychoeducation developed initially for patients with mental health problems, particularly schizophrenia, as experts acknowledged their need for an appropriate family environment (a relevant factor influencing patients clinical status), as well as family members’ needs and rights to get information and training on how to cope with the illness of their beloved. Progressively, psychoeducational programs also developed for other mental health conditions, and eventually for physical illnesses demanding significant changes in the patients’ life, such as cancer, HIV, epilepsy, and chronic pain conditions as FM [24]. In contrast to traditional medical models focused on symptom amelioration by means of pharmacological treatment, psychoeducation comes forward as a paradigm shift, aiming at competency, stressing health collaboration, coping and empowerment. Psychoeducation assumes that the more knowledgeable the patients are the more positive health-related outcomes will be. The fundamental psychoeducational model incorporates both illness-specific information and tools for managing related life challenges [47]. Overall, the selected papers in this review presented programs including education on FM, regarding its primary symptoms and recommended treatments. From the psychological perspective, the programs covered topics on emotional states and their association with symptoms, providing training on skills to promote relaxation and behavioral change, particularly from the CBT and the MBSR approaches. Yet, the organization of content was slightly different, some projects merged psychoeducational sessions and CBT in a single program, alone [35] or with other non-pharmacological alternatives [34,36], while other projects proposed psychoeducational programs that included topics on autogenic relaxation and/or MBSR [38,39,40,41,42,43]. The number and duration of the psychoeducational interventions were similar, including 8 or 9 sessions, lasting from 8 to 11 weeks. There is no consensus about the idoneous number of sessions, and this is more confusing as in many cases the name of psychoeducation is applied to simple educational interventions [24]. Moreover, the content of the program must be adapted to the particular illness shared by participants, and consider their particular features (e.g., age, presence of cognitive deficit) and life conditions (e.g., mobility, time to attend). Furthermore, research suggests that there are three distinct FM patient subgroups: One exhibiting extreme tenderness but lack any associated psychological/cognitive factors; an intermediate group with moderate tenderness and normal mood; and a third group with symptoms highly influenced by mood and cognitive factors [48]. Therapists must take into account these clinical features for a personalized approach.

Pain was the outcome measure common to all the studies, with an overall significant decrement in all groups receiving psychoeducation. A recent review [19] regarding the use of psychotherapy for physical pain in patients with FM found that CBT and imagery were the most commonly used approaches. Although improvements on physical pain were reported on all studies, results were not lasting and/or significant. In concurrence, this review found that CBT techniques are a common element in psychoeducational programs, probably due to evidence of its effectiveness, particularly in pain relief, improvement of health related quality of life, and reducing negative mood, disability, and fatigue [27]. In addition to pain, the studies also reported improvements in other physical symptoms such as fatigue, stiffness, and sleep quality. Emotional status was also benefited, as the results regarding depression, anxiety, stress, and cognitive impairment suggest. Also, daily functioning, health perception, social activity, and quality of life improved. In line with previous evidence [25], this research found that most of the considered studies reported positive results on patients’ condition, suggesting that an interdisciplinary intervention containing psychoeducation is a promising strategy in the patient-specialist management of FM. Regrettably, given the diversity of content in the psychoeducational programs, the outcome variables and their assessment instruments, an absolute effect to corroborate the effectiveness of psychoeducation to improve the clinical status of patients with FM could not be estimated.

Psychoeducation, having emerged from the mental health field, has progressively extended as a complementary treatment for illnesses that, due to their chronicity and/or the severity of the symptoms, require the patients’ involvement in long term. This review presented studies regarding psychoeducational programs specifically designed for patient with FM. Although the only author performed the bibliographical search, data collection and analyses, and that may be a source of bias, the procedure can be replicated following the steps presented in the method section. Moreover, in order to favor objectivity, the quality of the studies was assessed by two independent raters. One difficulty when searching for psychoeducational programs is that there is no homogeneity regarding their content, and even some programs covering only the educational or the therapeutic dimension are wrongly named psychoeducation.

## 5. Conclusions

The results from the present systematic review on psychoeducational programs for patients with FM show not only that they are feasible, but also that they can be effective for managing physical and emotional symptoms, in particular, pain and depression. As required, all psychoeducational interventions integrate information on FM and training for emotional coping and psychotherapeutic support, mainly from the CBT approach. In order to assess adequately the overall effectiveness of the programs it would be necessary that the studies include programs equivalent in content and use equal outcome measures.

## Figures and Tables

**Table 1:** Search queries by database.

Database	Date of Search	Query	Results
PubMed	13 October 2020	(fibromyalgia[Title/Abstract]) AND (psychoeducation[Title/Abstract])	7
Web of Science	13 October 2020	TOPIC: (fibromyalgia) AND TOPIC: (psychoeducation)	10
Scopus	13 October 2020	(TITLE-ABS-KEY (fibromyalgia) AND TITLE-ABS-KEY (psychoeducation))	38
Cochrane Library	13 October 2020	“fibromyalgia” in Title Abstract Keyword AND “psychoeducation” in Title Abstract Keyword	17
PsycInfo	13 October 2020	(fibromyalgia and psychoeducation).ti. fibromyalgia.ti. and psychoeducation.ab. (fibromyalgia and psychoeducation).ab. fibromyalgia.ab. and psychoeducation.ti.	5

**Table 2:** Basic features of publications.

Reference	Setting	Funding	Design	Sample Size	% of Females	Age Inclusion Criterion	Sample Mean Age	Assessment Time Points
**Original Research**								
[34]	USA	NR	Two-arm RCT	98	91.8	NR	52.06	Before/After treatment
[35]	USA	NR	Three-arm RCT	58	93	18–65	46.59	Before/After treatment 21-week follow-up
[36]	Canada	Yes	Two-arm RCT (wait list)	58	92.9	≥18	48.36	Before/After treatment 3-month follow-up
[37]	USA	Yes	Single-arm pre-post	67	88	8–20	13.9	Before/After treatment
[38] *	Spain	Yes	Two-arm RCT	216	97.65	18–75	55.3	Before/After treatment
[39] *	Spain	Yes	Two-arm RCT	216	97.65	18–75	55.3	Before treatment 12-month follow-up
[40] **	Spain	Yes	Single-arm pre-post (RCT pilot)	35	97.1	NR	54.97	Before/After treatment
[41] **	Spain	Yes	Three-arm RCT	225	98.23	18–65	53.27	Before/After treatment 12-month follow-up
**Study Protocol**								
[42] **	Spain	Yes	Three-arm RCT	180	-	18–65	-	Before/After treatment 12-month follow-up
[43] *	Spain	Yes	Two-arm RCT	218	-	18–75	-	Before treatment 1, 2, 6, and 12 months after the beginning of the intervention

Notes. References are presented alphabetically by the name of authors. USA: United States of America. NR: not reported. RCT: randomized control trial. * Publications from a common research project (FibrolQoL study) performed in three primary health care centers located in Viladecans-2, Gavà-1 and Gavà-2, all in Catalonia. ** Publications from a common research project (EUDAIMON study) performed in one single hospital located in St. Boi de Llobregat, Catalonia.

**Table 3:** Treatment groups, instruments, and significant results in the patients’ clinical outcome.

	Original Research
[34]	Groups. (1) Pharmacological treatment, *n* = 20, (2) pharmacological and non-pharmacological treatment, *n* = 78
	Instruments: Fibromyalgia Impact Questionnaire (20 items), The Multiaxial Diagnostic Inventory–Revised, Short Form 36 Health Survey, Visual Analogue Scale (0 to 10), Number of tender points. Outcome. A significant decrease in measures of function, chronic depression, general anxiety, somatization, pain, anxiety, and tender points for the group 2. Also a significant increase in the patients’ perceived overall better health. No significant changes in the control group. Effect size values: Not reported.
[35]	Groups. (1) Cognitive behavioral therapy and milnacipran, *n* = 20, (2) Education and milnacipran, *n* = 19, (3) Cognitive behavioral therapy and placebo, *n* = 19
	Instruments: Wrist-watch pain monitor (ActiWatch), Short Form 36 Health Survey Physical Functioning Subscale, Fibromyalgia Impact Questionnaire, Patient Health Questionnaire 8-item Depression Scale, Thumb Pressure Pain Sensitivity Test. Outcome. Compared with group 2, group 1 improved in physical function and in reduced pain intensity. Compared with group 2, group 3 had a moderate to large effect in improving physical function. Effect size values: Group 1, compared to group 2, showed improvement on physical function (0.60) and a reduction in pain intensity (0.67). Group 3, compared to group 2, showed improvement in physical function (0.70). Group 1, compared to group 2, showed improvement in physical function (0.60) and in reducing pain intensity (0.67). Group 1, compared to group 2, showed no significant differences in neither pain intensity (0.27) or physical function (0.10). Group 3, compared to group 2, showed little improvement in physical function (0.70) and no significant difference in pain intensity (0.40). The effect sizes for all the pair-wise group comparisons were very small (<0.30) regarding disease impact, depression severity, and changes in pain Sensitivity.
[36]	Groups. (1) Intervention, *n* = 29, (2) Wait list, *n* = 29 Instruments: Numerical rating scale for pain sensitivity (0 to 10), Fibromyalgia Impact Questionnaire (20 items), Modified Brief Pain Inventory, Chronic Pain Sleep Inventory, Coping Strategy Questionnaire, Beck Depression Inventory, Short Form 12 Health Survey, Global Impression of Change, Pain Relief Scale (0 to 100).
	Outcome. Group 1 significantly improved in perceived pain levels, pain relief and quality of life. Effect size values: Intervention group showed improvement after treatment (−0.13) and 3 months later in perceived pain levels (−0.55). Intervention group showed improvement after treatment (−0.36) and 3 months later in pain relief (−0.64). Intervention group showed improvement after treatment (1.14) and 3 months later in quality of life (1.84).
[37]	Groups. (1) Intervention, *n* = 67
	Instruments: Brief Symptom Inventory, Visual Analogue Scale of the Pediatric Pain Questionnaire, Pittsburgh Sleep Quality Index, Fatigue Severity Scale, Functional Disability Inventory, Multidimensional Anxiety Scale, Children’s Somatization Inventory, Child Behavior Checklist, Satisfaction with Abilities and Well-Being Scale. Outcome. Significant reductions in pain, somatic symptoms, anxiety, and fatigue. Significant improvements in sleep quality, functional ability and fewer school absences. Effect size values: Not reported.
[38]	Groups. (1) Psychoeducation and usual care, *n* = 108, (2) Usual care, *n* = 108 Instruments: Fibromyalgia Impact Questionnaire (Spanish Version, 10 items), Chronic Medical Conditions Checklist, State Trait Anxiety Inventory (Spanish Version), Marlowe-Crowne Social Desirability Scale.
	Outcome. Group 1 improved in functional status, physical impairment, days not feeling well, pain, general fatigue, morning fatigue, stiffness, anxiety, and depression. Effect size values: functional status (0.16), physical impairment (0.09), days not feeling well (0.08), pain (0.12), general fatigue (0.04), morning fatigue (0.05), stiffness (0.03), anxiety (0.08), depression (0.09).
[39]	Groups. (1) Psychoeducation and usual care, *n* = 108, (2) Usual care, *n* = 108 Instruments: Chronic Medical Conditions Checklist, Fibromyalgia Impact Questionnaire (10 items), EuroQoL-5D questionnaire, Client Service Receipt Inventory—Adapted.
	Outcome. Group 1 showed greater improvement in global functional status, physical functioning, days feeling well, pain, morning fatigue, stiffness, and depression. Effect size values: Global functional status (0.36), physical functioning (0.56), days feeling well (0.40), pain (0.35), morning fatigue (0.24), stiffness (0.34), depression (0.30).
[40]	Groups. (1) Intervention, *n* = 35
	Instruments: Humor Styles Questionnaire, Five-Facets Mindfulness Questionnaire, Patient Global Impression of Change (PGIC), Revised Fibromyalgia Impact Questionnaire (21 items). Outcome. Improvement in the perceived impact of FM, and perceived changes in observed mood, social activity, over-all status, physical activity, work activity, and pain. Effect size values: Not reported.
[41]	Groups. (1) MBSR and treatment as usual, *n* = 75, (2) FibroQoL and treatment as usual, *n* = 75, (3) treatment as usual, *n* = 75
	Instruments: Revised Fibromyalgia Impact Questionnaire (21 items), Fibromyalgia Survey Diagnostic Criteria, Hospital Anxiety and Depression Scale, Pain Catastrophizing Scale, Perceived Stress Scale, Multidimensional Inventory of Subjective Cognitive Impairment, Five Facets of Mindfulness Questionnaire, Self-Compassion Scale—short form, Psychological Inflexibility in Pain Scale, Patient Global Impression of Change, Pain-Specific Impression of Change, Credibility/Expectancy Questionnaire, daily logs for recording the frequency of practice of MBSR exercises during the intervention. Outcome. Group 1 was superior to group 2 at post-treatment in measures of functional impact, anxiety and depression, perceived stress, and cognitive impairment, and at post-treatment and follow-up in measures of pain catastrophizing. Group 1 was superior to group 3 both at post-treatment and at follow-up in measures of functional impact of FM, symptoms of FM, anxiety and depression, pain catastrophizing, perceived stress, and cognitive impairment. Group 2 was superior to group 3 at post-treatment in measures of symptoms of FM, and at follow-up in measures of anxiety and depression, and cognitive impairment. Effect size values: Group 1 compared to group 2 at post-treatment in measures of functional impact (0.86), anxiety and depression (0.49), perceived stress (0.77), and cognitive impairment (0.95), and at post-treatment and follow-up in measures of pain catastrophizing (0.65, 0.58). Group 1 compared to group 3 at post-treatment and at follow-up in measures of functional impact of FM (1.11, 0.80), symptoms of FM (0.97, 1.04), and cognitive impairment (0.86, 0.99). Group 2 was superior to group 3 at post-treatment in measures of symptoms of FM (0.54) and at follow-up in measures of anxiety and depression (0.57), and cognitive impairment (0.65).
	**Study Protocol**
[42]	Groups. (1) Mindfulness-Based Stress Reduction and treatment as usual, *n* = 60, (2) FibroQoL and treatment as usual, *n* = 60, (3) treatment as usual, *n* = 60 Instruments: SCID-I Structured Clinical Interview for DSM Axis I Disorders—Depression module, Mini-Mental State Examination, Revised Fibromyalgia Impact Questionnaire, Fibromyalgia Survey Diagnostic Criteria, Hospital Anxiety and Depression Scale, Perceived Stress Scale, Multidimensional Inventory of Subjective Cognitive Impairment, EuroQoL-5D questionnaire, CSRI Client Service Receipt Inventory, Five Facet Mindfulness Questionnaire, Pain Catastrophizing Scale, Psychological Inflexibility in Pain Scale, Self-Compassion Scale, Structural and Functional Neuroimaging data, Inflammatory data, Credibility/Expectancy Questionnaire, Patient Global Impression of Change, Patients Specific Impression of Change.
[43]	Groups. (1) Psychoeducation and usual care, *n* = 109, (2) Usual care, *n* = 109 Instruments: Questionnaire on chronic medical condition, Fibromyalgia Impact Questionnaire (Spanish Version, 10 items), EuroQoL-5D questionnaire, Client Service Receipt Inventory—Adapted.

**Table 4:** Outline of the interventions.

Ref. [34] Non-pharmacologic treatment included cognitive behavioral therapy, coping skills classes, pool or land exercise, massage therapy, auricular therapy, microcurrent therapy, and nutritional counselling. The intervention lasted for approximately one year. Psychoeducation/cognitive behavioral therapy sessions were part of the phase 1 of the program, lasting eight weeks.
Ref. [35] Eight sessions of psychoeducation/cognitive behavioral therapy: (1) education on chronic pain including theories of pain; (2) training in progressive muscle relaxation and visual imagery; (3) education on the relationship between automatic thoughts and pain; (4) cognitive restructuring; (5) stress management; (6) time-based pacing and pleasant activity scheduling; (7) anger management and sleep hygiene; and (8) relapse prevention and flare-up planning.
Ref. [36] The PASSAGE program includes 9 sessions covering three dimensions: (1) psychoeducational tools, (2) cognitive behavioral therapy techniques, and (3) exercise activities. Psychoeducational topics: (1) introduction, (2) FM symptoms, (3) exercise and physical activity, (4) psychological state, (5) energy and capacity, (6) the vicious circle of chronic pain, (7) pharmacological and non-pharmacological treatment, (8) review and summary, (9) follow-up visit.
Ref. [37] Intervention for children and their parents, with four primary treatment modules lasting 8 weeks. (1) Psychoeducation (symptoms, etiology, treatments), week 1, (2) sleep improvement, weeks 2–3, (3) pain management, weeks 4–6, (4) activities of daily livings, weeks 7–8.
Refs. [38,39,43] The intervention consisted of 9 sessions over a 2-month period, intercalating 5 educational sessions with 4 autogenic training sessions. The educative sessions included information about typical symptoms, usual course, comorbid medical conditions, potential causes of the illness, the influence of psychosocial factors on pain, current pharmacologic and nonpharmacologic treatments, the benefits of regular exercise, and the typical barriers to behavior change. The autogenic training sessions focused on immediate physical and mental relaxation, pain relief, and stress reduction,
Ref. [40] The MINDSET (MINDfulneSs & EducaTion) program included intercalated four sessions of psychoeducation and four sessions of mindfulness training. Education sessions focused on: (1) overview of FM, (2) the pillars of good health, (3) emotion management and communication skills, (4) services and resources. Mindfulness sessions focused on: (1) mindfulness and chronic pain, (2) focus on the breath, thoughts and emotions, (3) management of unpleasant experiences, (4) action plan for the daily use of mindfulness.
Refs. [41,42] The eight Mindfulness-Based Stress Reduction sessions introduced and practiced techniques to change the reaction to pain: the raisin-eating task, the inquiry, and the body scan, conscious movements, the 9-point exercise, sitting meditation, walking meditation, and the metta exercise. The eight FibroQoL sessions focused on (1) updated medical information on FM, (2) physical and emotional pain, (3) diagnosis and treatment, (4) personal goals and obstacles, (5) self-esteem and emotions, (6) plans for personal changes, (7) review of achievements, and (8) the role of specialized care.

## Data Availability

The dataset generated and/or analyzed during the current study is available from the corresponding author on reasonable request.
